# The Medical, Surgical, and Hygienic Exhibition

**Published:** 1904-06-04

**Authors:** 


### THE MEDICAL, SURGICAL, AND HYGIENIC EXHIBITION.

THE increased patronage which the medical profession extends to this exhibition year by year and the absorbing interest which it affords substantiate beyond question its claim to provide a convenient and easily accessible means of investigating the most advanced and improved forms of appliances, antiseptics, drugs, dietetic and other food preparations which are from time to time produced for medical and nursing purposes. The exhibition which was held last week for four days commencing May 24th was, in point of numbers and class of attendance, more successful than its predecessors; but there were fewer exhibits, probably because the aim of the authorities is to present only that which is of medical, nursing and institutional interest. The progress which has been made in the therapeutics of light and heat was demonstrated by the Dowsing Radiant Heat Company in their baths and apparatus for the application of electric light and heat rays in the treatment of disease, and particular mention should be made of a most convenient cabinet for giving an electric-light bath at home.

There were numerous examples of infant, invalid, and diabetic foods and dietetic preparations. An exhibit of interest was Billon's Ovo Lecithin, a distearo-glycerophosphate of choline prepared from the yolk of egg; as was also Messrs. W. A. Jones and Co.'s special and highly nutritious preparation of the banana called Bananine. The fluid beef "Oxo" (Liebig's Extract of Meat Company) engaged much attention, and Cerebos Salt, which is now so largely used, was prominent. The Shredded Wheat Company exhibited their whole-wheat preparations, including "Triscuit," whilst Cadbury's cocoa and chocolate were much favoured on account of their excellence. Bovril in various forms, and "Virol," the well-known red bone marrow food, were much in evidence, as was also "Marmite," a nutritious and highly concentrated vegetable extract eminently suitable for invalids and dyspeptics. Diabetic foods were shown by the Protene Company, Limited, who exhibited several new forms of palatable preparations, including their bread flour for home baking; the Manhu Food Company with Manhu diabetic specialities, Messrs. Callard and Co., whose diabetic fruits, jellies, etc., created interest, and Messrs. Van Abbott and Sons, whose gluten bread, biscuits, etc. are well known. Messrs. Mellin's Food, Limited, exhibited their food for infants and invalids, also Lacto-Glycose, which they recommend where fresh cow's milk cannot be relied upon. "Viking Milk," a condensed milk without sugar, was exhibited by Henri Nestlé, together with Nestlé's food for infants and invalids, and condensed milk. The exhibit of the Anglo-Swiss Milk Company, which included their "Ideal" milk, is worthy of notice; also that of the Galak Milk Products Company with dried milk products in powder and tablet form. International Plasmon, Limited, exhibited many forms of Plasmon, including Plasmon chocolate, and Plasmon arrowroot, while demonstrations in making Plasmon whipped cream were given at frequent intervals. Among other exhibits may be mentioned Hovis Bread and Flour, Robinson's Patent Barley and Groats (Messrs. Keen, Robinson, and Co., Limited), and a preparation of eggs and milk extract of much nutritive value, called Maltova.

Messrs. W. R. Warner and Co.'s pharmaceutical preparations were exhibited by Messrs. J. Newbery and Sons, and included Tono-Sumbul, Ingluvin, and "Parvules," or minimum doses of active substances for frequent administration. Messrs. Burgoyne, Burbidges, and Co. provided an exhibit of Messrs. Von Heyden and Co.'s products, which attracted marked interest, and which included Collargolum, for application in septic diseases; Creosotal, for administration in pulmonary and bronchial affections; Acoine, a local anæsthetic, and many other preparations. The Denver Chemical Manufacturing Company's Antiphlogistine, an antiseptic absorbent for external application in inflammatory conditions, received considerable notice. The Angier Chemical Company showed their well-known emulsion, also a new throat tablet; whilst the principal exhibit of the Peptenzyme Company was a preparation named Peptenzyme, which has been found by investigation to be an effective digestant. An exhibit of interest was that of the Maltine Manufacturing Company, Limited, who displayed their excellent maltine preparations and Carnrick's liquid peptonoids, prepared from beef, wheat, and milk. Somnoform, an anæsthetic which is asserted to be quick and safe in its effect, afforded considerable interest; and the Sanitary Wood Wool Company's display of Hartmann's antiseptic wood wool preparations was also much noticed.

The Peat Products Company exhibited hitherto little known antiseptic soap preparations bearing the distinctive name of Sphagnol, the active agents in which are vegetable tars and oils obtained from peat, and which claim to be of much value in skin affections, and particularly useful in tropical countries. Much attention was devoted to the Izal disinfectants which are so much in medical and public favour, and have been largely supplied for the plague in South Africa, while Pearson's antiseptics also received considerable notice.

"Arabella" water, for use in cases of gout, obesity, and liver troubles, was much in evidence, while Messrs. Ingram and Royle's exhibit of Vichy and Carlsbad waters afforded considerable interest. Table waters were shown by Camwal Limited and by Idris and Co., Limited, who had a patent syphon with a removable porcelain top, which renders metallic contamination impossible. Messrs. Cooper and Co. also exhibited under this head, with "Globenaris" waters, contained in elegant porcelain-lined syphons.

With regard to publications, the Scientific Press, Limited, exhibited a number of medical and nursing works, foremost amongst which were a new and exhaustive text-book on midwifery, by Dr. J. K. Watson, and a case book for private nursing, both of which commanded much attention from nurses. Medical publishers were also well represented by Mr. Sidney Appleton and Messrs. Rebman Limited.

